# FcRn as a Transporter for Nasal Delivery of Biologics: A Systematic Review

**DOI:** 10.3390/ijms22126475

**Published:** 2021-06-17

**Authors:** Maxime Fieux, Sandra Le Quellec, Sophie Bartier, André Coste, Bruno Louis, Caroline Giroudon, Mikail Nourredine, Emilie Bequignon

**Affiliations:** 1 Hospices Civils de Lyon, Centre Hospitalier Lyon Sud, Service d’ORL, D’otoneurochirurgie et de Chirurgie Cervico-Faciale, Pierre Bénite, CEDEX, F-69495 Lyon, France; 2 Université de Lyon, Université Lyon 1, F-69003 Lyon, France; sandra.le-quellec@chu-lyon.fr (S.L.Q.); mikail.nourredine@chu-lyon.fr (M.N.); 3 Univ Paris Est Creteil, INSERM, IMRB, F-94010 Créteil, France; sophiebartier@hotmail.fr (S.B.); andre.coste@chicreteil.fr (A.C.); bruno.louis@inserm.fr (B.L.); emilie.bequignon@gmail.com (E.B.); 4 CNRS ERL 7000, F-94010 Créteil, France; 5 Hospices Civils de Lyon, Hôpital Cardiologique Louis Pradel, Unité D’hémostase Clinique, CEDEX, F-69500 Bron, France; 6 EA 4609 Hémostase et Cancer, Université Claude Bernard Lyon 1, F-69372 Lyon, France; 7 Hospices Civils de Lyon, Centre de Biologie et de Pathologie Est, Service D’hématologie Biologique, CEDEX, F-69500 Bron, France; 8 Service d’ORL, de Chirurgie Cervico Faciale, Hôpital Henri Mondor, Assistance Publique des Hôpitaux de Paris, F-94000 Créteil, France; 9 Service d’ORL, de Chirurgie Cervico Faciale, Centre Hospitalier Intercommunal de Créteil, F-94010 Créteil, France; 10 Hospices Civils de Lyon, Service de la Documentation Centrale, CEDEX, F-69424 Lyon, France; caroline.giroudon@chu-lyon.fr; 11 Hospices Civils de Lyon, Service de Biostatistique et Bioinformatique, F-69003 Lyon, France; 12 CNRS, Laboratoire de Biométrie et Biologie Évolutive UMR 5558, F-69100 Villeurbanne, France

**Keywords:** neonatal Fc receptor, monoclonal antibody, immunoglobulin G, Fc-fusion protein, transcytosis, nasal route, biologics

## Abstract

FcRn plays a major role in regulating immune homeostasis, but it is also able to transport biologics across cellular barriers. The question of whether FcRn could be an efficient transporter of biologics across the nasal epithelial barrier is of particular interest, as it would allow a less invasive strategy for the administration of biologics in comparison to subcutaneous, intramuscular or intravenous administrations, which are often used in clinical practice. A focused systematic review was conducted following the Preferred Reporting Items for Systematic Reviews and Meta-Analyses (PRISMA) guidelines. It was registered on the international prospective register of systematic reviews PROSPERO, which helped in identifying articles that met the inclusion criteria. Clinical and preclinical studies involving FcRn and the nasal delivery of biologics were screened, and the risk of bias was assessed across studies using the Oral Health Assessment Tool (OHAT). Among the 12 studies finally included in this systematic review (out of the 758 studies screened), 11 demonstrated efficient transcytosis of biologics through the nasal epithelium. Only three studies evaluated the potential toxicity of biologics’ intranasal delivery, and they all showed that it was safe. This systematic review confirmed that FcRn is expressed in the nasal airway and the olfactory epithelium, and that FcRn may play a role in IgG and/or IgG-derived molecule-transcytosis across the airway epithelium. However, additional research is needed to better characterize the pharmacokinetic and pharmacodynamic properties of biologics after their intranasal delivery.

## 1. Introduction

The neonatal receptor for the Fc fragment of immunoglobulin (Ig) G (FcRn) was discovered in the 1980s [1]. It is a transporter of maternal immunoglobulin G (IgG) to the foetus during gestation (humans, rodents), as well as through breast milk during the neonatal period (rodents) [2,3,4,5]. However, more recent studies have demonstrated that FcRn is rather ubiquitous and is expressed throughout life [6,7,8]. FcRn is expressed in endothelial cells and haematopoietic cells, as well as in airway epithelial cells, including human nasal epithelial cells (HNECs) [9,10], alveolar epithelial cells [11,12,13,14] and porcine olfactory mucosa [15,16]. FcRn plays a major role in regulating immune homeostasis in adults and in regulating the half-life of IgG by protecting it from degradation using a recycling mechanism [17,18,19]. Moreover, FcRn impacts the biodistribution of IgG-like monoclonal antibodies (mAbs); it transports antibodies across the cellular barrier [6,7,8,11,12,20,21,22,23,24,25,26] and delivers IgGs across mucosal surfaces to confer protective immunity [27,28,29,30,31,32,33]. The role of FcRn in the transport of molecules in epithelial cells has been extensively analyzed [11,12,22,23,24,25,34,35]. IgG binding to FcRn occurs with the highest affinity at the acidic pH (pH 5~6) found in endosomes, and with the lowest affinity at neutral pH (pH 7.4), thereby allowing for cell-surface dissociation in the plasma [36,37]. Interestingly, a pH of approximately 6 is also found at the apical cell surface of various mucosal layers [38]. More recently, it has been shown that FcRn also regulates the homeostasis of albumin [39]. Of note, the binding site for albumin on FcRn is different from the one for IgG [40,41]; hence, they do not compete with each other for FcRn binding.

Since the introduction of hybridoma technology by Köhler and Milstein, some of the most important biologics that have been developed are IgG-derived antibodies (Abs) [42,43]. FcRn-mediated transport can be used for the delivery of therapeutic Abs or other types of biologic following various administration routes [44,45]. In 2012, a study showed that bevacizumab (Avastin^®^; Roche, Bâle, Suisse) was able to cross two subtypes of porcine mucosa: one mucosa obtained from the septum and the other one obtained from the snout cavity. The author supposed this was due to FcRn-mediated transport of IgG-derived molecules through the epithelium [46]. This study opened the door for the use of intranasal delivery of biologics. However, although FcRn expression and its role in IgG transport were characterized in the lower airways in the early 2000s [11,12,13,14,47], FcRn expression in the upper airways has not yet been clearly identified [10]. Additional studies are required to identify the directionality of FcRn-mediated IgG transport in the nasal epithelium. In humans, intranasal delivery of insulin has been shown to induce a positive effect on memory and metabolic effects via the hypothalamic–pituitary axis [48,49,50,51,52]. Whether the nose-to-brain pathway is also navigable for proteins with a higher molecular weight, such as Abs, remains to be determined. Balin et al. demonstrated that horseradish peroxidase was detectable in the olfactory bulb of rodents and monkeys within 45 to 90 min after intranasal delivery [53], thus suggesting that axons of olfactory neurons may represent a pathway to the brain for protein drugs such as Abs [54]. However, several questions remain to be addressed with regard to the intranasal delivery of biologics. Although it has been shown that biologics are rapidly efficient after intranasal delivery [55], their bioavailability has not yet been completely solved. In addition, the generation of micron-sized aerosol droplets can have tremendous effects on proteins. The shear stress occurring during aerosol formation can induce the formation of unfolded proteins or protein aggregation [56,57,58,59,60], with a subsequent decrease in biological efficiency and immune side effects [61]. Therefore, it is of great importance to further study the behaviour of biologics after their intranasal delivery in various preclinical models.

The current review will be the first to specifically discuss the use of FcRn in the transcytosis of biologics after intranasal delivery. This review is a prerequisite to further developments of the nasal route for the administration of biologics, which can considerably improve patients’ quality of life, especially in those who undergo multiple lifelong intravenous and/or subcutaneous injections for the treatment of chronic diseases.

To address this question, we hereby provide a systematic review of the implications of FcRn in the transport of biologics after intranasal delivery. Systematic reviews tend to be more comprehensive and less biased than other types of literature reviews. They are supposed to be built on a defined protocol that describes the rationale, the hypothesis and the planned method used for drawing theoretical conclusions from the various studies of interest. Therefore, systematic reviews have become an increasingly central pillar of basic science and represent a substantive contribution to knowledge [62], especially as the number of publications increases overtime. To increase the transparency of our review, it was registered in the international prospective register of systematic reviews PROSPERO (CRD42021236019, https://www.crd.york.ac.uk/prospero/#recordDetails, accessed on 8 March 2021), following the Preferred Reporting Items for Systematic Reviews and Meta-Analyses (PRISMA) guidelines [63,64]. In addition, the risk of bias for each study selected was assessed using the Oral Health Assessment Tool (OHAT) [65,66], which has been developed for evaluating the internal validity of studies [65,66,67].

In this review, the recorded outcomes were the effects of biologics after nasal administration following FcRn-mediated transcytosis across the epithelium. In vivo studies were more specifically investigated for pharmacokinetic (absorption, distribution, metabolism, elimination, transcytosis efficiency, time and plateau effect), pharmacodynamic (dose-effect response and drug activity) and toxicity (cellular viability, epithelial permeability, cell differentiation, ciliary analysis) outcomes. In vitro studies were investigated for pharmacokinetic (transcytosis efficiency, time effect, plateau effect and repeated administration effect), pharmacodynamic (dose-effect response and drug activity) and toxicity (cellular viability, epithelial permeability, cell differentiation and ciliary analysis) outcomes. Studies reporting any other types of outcome were excluded.

## 2. Methods

This systematic literature review was conducted and reported in line with criteria stipulated by the PRISMA recommendations [63,64]. Literature monitoring was performed regularly until 25 April 2021. Our eligibility criteria included clinical, in vivo and in vitro preclinical studies involving FcRn and nasal delivery of any type of biologic. Eligible articles were original research articles irrespective of their publication date. Clinical studies that were considered for eligibility were randomized or nonrandomized controlled clinical trials, comparative studies and observational studies. Animal and in vitro preclinical studies had to be relevant to human health. Systematic reviews, narrative reviews and single-patient case reports were excluded.

Studies were identified by searching electronic databases such as PubMed (including Medline, the US National Library of Medicine), the Cochrane Library (Wiley), Web of Science (Clarivate Analytics) and Scopus (Elsevier). Additional sources obtained from the reference lists of selected articles were identified. Only English and French language articles were included.

**Figure 1:**
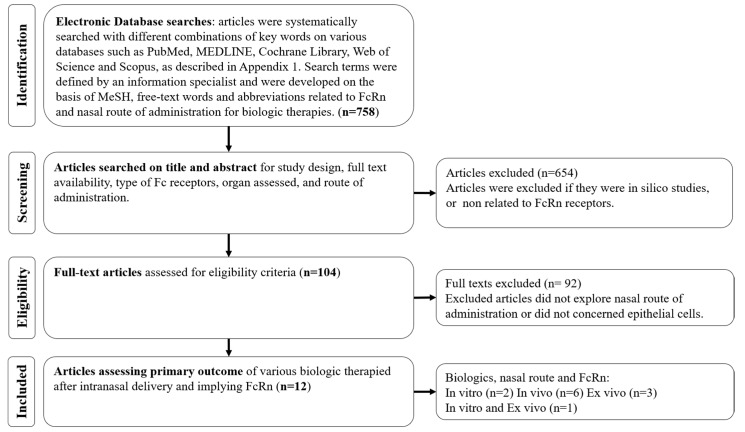
PRISMA Study Selection Flow Diagram. The PRISMA Flow Diagram demonstrates each phase of the search strategy in which articles were evaluated. During each phase, articles were excluded based on our eligibility criteria. Bold numbers correspond to the number of articles selected after each phase of the search strategy.

The keywords included and the detailed equations defined by an information specialist from the Lyon University Hospitals Documentation Centre are given in the Supplementary Materials (Supplementary File S1). Study selection was performed as described, using the PRISMA flow diagram (Figure 1) [63], and was conducted using the program Rayyan (Rayyan Systems Inc., Cambridge, MA, USA) [68]. The data were collected (title, author, year, journal, number of references, study type, species, intervention, comparison group, assessment, outcome and risk of bias) and presented in a standardized Microsoft Excel^®^ form developed for this systematic review.

The risk of bias was assessed across studies using the OHAT (Supplementary File S2, [65,66,67]). The OHAT assessed bias in the studies using 11 questions that target selection bias, cofounding bias, performance bias, attrition bias, detection bias and selective reporting bias. Only 9 questions were used to assess the bias of in vitro exposure and experimental animal studies. Each question was answered by one out of four levels: definitely low, probably low, probably high and definitely high risk of bias.

The primary outcomes of interest were the effects of biologics after nasal administration and the role of FcRn in epithelial transcytosis within the upper airway system. The results were synthesized based on the reported measures from the included studies. Performing a meta-analysis was impossible because there were no trials using a similar methodology or reporting a similar outcome.

## 3. Results

### 3.1. Selection, Characteristics and Risk of Bias of the Included Studies

Utilizing our inclusion and exclusion criteria, 12 articles were included [9,10,15,16,26,27,28,29,35,46,61,69]. Among these, an in vitro HNEC model was used in one study [9], an in vitro Roswell Park Memorial Institute (RPMI) epithelial model was used in two studies [16,35], an in vivo murine model was used in six studies [26,27,28,29,61,69], an ex vivo porcine model was used in 3 studies [15,16,46] and an ex vivo human model was selected in one study [10]. Table 1 describes the general characteristics of the included studies. Additional details about the in vivo and in vitro studies are described in the Supplementary Materials in Supplmentary Files S3 and S4, respectively.

The risk of bias was assessed using the OHAT, even though we analyzed in vivo and in vitro studies (Table 2). The majority of studies using animal models [15,16,26,27,28,29,46,61,69] investigated the ability of FcRn to enhance transcytosis (*n* = 8, [15,16,26,27,28,29,61,69]), while only two investigated the epithelial toxicity of this procedure (*n* = 2, [26,46]). In studies performed on human cells [9,10,16,35], three considered transcytosis efficiency [9,16,35] and one considered transcytosis toxicity [9].

### 3.2. Expression of FcRn in the Upper Airway System

Among the 12 studies included in this systematic review, six first confirmed the expression of FcRn in their different models chosen to assess biologic-transcytosis across airway epithelial cells [9,10,15,16,28,29]. Several methods were used to study FcRn expression. Most mouse-based studies assessed FcRn expression in various tissues using immunohistochemical analyses. Mouse FcRn was found to be expressed in the lungs and trachea [28,29] but not in the intestine of adult mice [29]. The expression pattern of FcRn in larger animal models and humans was studied using FcRn mRNA or FcRn protein measurements in cell cultures derived from in vivo tissue extraction [9,10,15,16]. In 2018, Ladel et al. showed that FcRn was expressed in different parts of the porcine regioolfactoria (*concha nasalis dorsalis*, *concha nasalis media*, *ethmoidal turbinates*), as well as in the porcine respiratory epithelium (*concha nasalis ventralis*) [15]. The transcription and expression of the *FCGRT* gene, encoding FcRn, was studied in OEPC and RPMI cells by RT-PCR and Western blots. The results were compared to those obtained from the porcine olfactory mucosa (*concha nasalis media*), which served as a reference [15,16]. There was a trend towards a lower expression level of FcRn in RPMI compared to OEPC [67]. In humans, Heidl et al. was the first to confirm the localization of the FcRn α-chain in ciliated cells of the epithelium in blood vessels and subepithelial glands using an affinity-purified antibody against the cytoplasmic tail of the FcRn α-chain in nasal tissue sections [10]. They showed that the steady-state distribution of FcRn was predominantly observed at the basolateral side of ciliated epithelial cells and gland cells. Colocalization of the FcRn α-chain with IgG or with early sorting endosomes (EEA1-positive), but not with late endosomes/lysosomes (LAMP-2-positive), in ciliated cells was observed. This was indicative of the presence of FcRn in the recycling/transcytosis pathway but not in compartments involved in lysosomal degradation, supporting the role of FcRn in IgG transcytosis in the nasal epithelium [10].

Bequignon et al. also demonstrated the expression of FcRn in HNECs. High expression of FcRn was found in the cytosol of ciliated, mucus and basal cells [9]. Interestingly, FcRn expression varies depending on the degree of cell differentiation [9].

The other six studies [26,27,35,46,61,69] did not specifically assess FcRn expression. Some of them used cell lines and mouse models that were already known to express FcRn [26,27,69], or the aim of the study was different [35]. In contrast, Samson et al. suggested that FcRn expression might explain bevacizumab permeability through the nasal epithelium, as it can bind IgG. However, FcRn expression in the nasal mucosa was not studied in this paper, and it had not been reported previously at that time [46]. More recently, Kumar et al. showed an AF488-IgG signal in the underlying lamina propria on high magnification confocal imaging of sections from the olfactory epithelium, but did not look at FcRn expression [61].

### 3.3. Biologics-Transcytosis Efficiency after Intranasal Delivery

Taken together, the combined expression of FcRn in the endothelium, glands and ciliated nasal epithelial and basal cells, as well as the localization of IgG in these tissues, suggests that FcRn could play a role in IgG transport in the nasal mucosa [10]. Among the 12 studies included in this systematic review, 11 demonstrated efficient transcytosis of biologics through the nasal epithelium [9,15,16,26,27,28,29,35,46,61,69]. The biologics that were studied were highly heterogeneous across the studies, as were their doses (Table 1). Most studies focused on immunization following intranasal delivery of biologics [27,28,29,69], whereas others aimed to characterize FcRn-mediated transport of biologics and their subsequent biodistribution [15,16,26,35,46,61]

Globally, all studies about immunization reported that intranasal delivery of biologics was efficient for inducing an appropriate immune response directed against the pathogen of interest. Some authors studied a fusion of inactivated *Francisella tularensis* (iFt) and IgG, named mAb-iFT, as a model for the Ft vaccine [27,69]. Rawool et al. demonstrated that (i) the FcR-targeted immunogen enhances immunogen-specific IgA production and protection against subsequent Ft infection in an IgA-dependent manner; (ii) both FcγR and FcRn are crucial to this protection; and (iii) iFt, when targeted to FcRs, enhances protection against the highly virulent SchuS4 strain of Ft, a category A biothreat agent. Bitsaktsis et al. demonstrated that FcRn targeting increased the frequency and activation status of DCs in the lungs of immunized mice. It mediates the generation of Ft-specific, gamma interferon (IFN-γ)-secreting, effector memory CD4^+^ T cells during infection, thus further elucidating the immunological mechanisms involved in enhanced immune protection utilizing this novel mucosal vaccine platform. Furthermore, 100% of the C57BL/6 mice that were immunized with mAb-iFT ICs survived the Ft LVS challenge, while immunization with iFT alone provided only 50% protection.

Other vaccine models were also tested. Gag-Fc fusion protein (fusion of p24 protein from HIV Gag with IgG heavy chain) was used for the HIV vaccine challenge model [28], and gD-Fc/wt (HSV-2 glycoprotein D fused with an IgG Fc fragment) was used to test the effect of glycosylation in the HSV vaccine challenge model [29]. Lu et al. demonstrated that the chimeric Gag-Fc fusion protein was transported efficiently across the mucosal epithelium in mice. Moreover, intranasal immunization induced an immune response sufficiently potent to protect mice from infection with HIV after the intravaginal challenge. Finally, FcRn-targeted immunization induced strong antibody and cellular immune responses to HIV Gag at mucosal and systemic sites [28]. Ye et al. demonstrated that the FcRn/IgG transport pathway can be exploited to greatly enhance the efficacy of mucosal-administered vaccines. The authors identified that FcRn-targeted mucosal immunization differs notably between WT and FcRn KO mice or between gD-Fc/wt and gD-Fc/mut immunized mice in terms of mucosal and systemic immune responses, cytokine expression profiles, the maintenance of T and B cell memory and long-lived bone marrow plasma cells, and the resistance to infection.

FcRn-mediated transcytosis after intranasal delivery was also studied in applications other than immunization. Radiolabelled Ab ([^125^I]-IgG) and fluorescently labelled Ab (AF488-IgG) were used to assess CNS delivery [61]. Kumar et al. demonstrated that intranasal [^125^I]-IgG consistently yielded the highest concentrations in the olfactory bulbs, trigeminal nerves and leptomeningeal blood vessels, as well as their associated perivascular spaces. Significantly higher [^125^I]-IgG concentrations were found in the CNS after intranasal delivery than after intra-arterial delivery for doses producing similar endpoint blood concentrations. Importantly, the concentration of [^125^I]-IgG in the CNS significantly increased in a dose-dependent manner following intranasal administration of increasing doses, from the picomolar range after administration of small doses (50 μg) up to the nanomolar range after administration of higher doses (1 mg and 2.5 mg). The authors showed that it might be feasible to achieve therapeutic levels of IgG in the CNS, especially when delivering high doses in the nose, and they provided insights about the nose-to-brain pathways the Abs took after intranasal delivery. As mentioned previously, IgG and albumin also bind to FcRn. In this context, Bern et al. assessed the intranasal delivery of wild-type (WT) albumin and engineered albumin, with a specific mutation responsible for a higher binding affinity for FcRn (KAHQ, QMP, and scFv-Alb) [26]. They demonstrated that approximately 25% of the WT albumin doses reached the blood circulation 24 h after intranasal delivery in Tg32 alb KO mice. This may correspond to 60 to 70% recovery in total if we consider that one-third of WT albumin is usually present in the blood and two-thirds is in the extravascular compartment. Accordingly, intranasal delivery to albumin-preloaded mice of scFv-WT and scFv-QMP showed that the resulting blood concentration of scFv-QMP was more than fourfold higher than the blood concentration of scFv-WT in Tg32 alb KO mice 24 h after administration. Maximum concentrations of scFv-QMP in sera were obtained at 8 h. The time to reach the maximum concentration of biologics may differ depending on biological properties themselves and the model used for pharmacokinetic assessment.

Bequignon et al. used an in vitro model of HNEC primary culture to study infliximab transcytosis. Infliximab is an IgG1 therapeutic mAb mostly used for the treatment of autoimmune disorders. The apical-to-basal experiment demonstrated effective and dose-dependent infliximab transfer across the HNECs on both day 7 and day 21 of cell differentiation.

Samson et al. demonstrated the transmucosal transport and bioavailability of bevacizumab [46]. Bevacizumab is an IgG1 therapeutic mAb directed against the vascular endothelial growth factor, and is mostly used for the treatment of some cancers. After intranasal delivery of bevacizumab in porcine olfactory mucosa, the total recovery of bevacizumab throughout the 2.5 h experiment was 83%. Histopathological analyses revealed that bevacizumab was distributed at the mucosal surface (53%), intracellularly (19%) and throughout the nasal mucosa (11%).

Finally, Ladel et al. in 2018 demonstrated the potential FcRn-mediated transport of epithelial and basal cells towards the lamina propria, facilitating the apical uptake of allogenic and, in lesser amounts, xenogenic IgG [15]. A year later, they also demonstrated that only traces of porcine IgGs (pIgGs) could be recovered at the basolateral compartment in ex vivo olfactory tissue, while human IgGs (hIgGs) reached far higher levels [16]. They also demonstrated comparable permeation rates for human and porcine IgG in primary cells from porcine olfactory epithelium (OEPC), which displayed the highest expression of FcRn. Nevertheless, at early time points in OEPC ALI cultures, the permeation of the hIgGs was significantly faster than that of the pIgGs [16].

### 3.4. Factors Influencing Transcytosis

It seems easily understandable that FcRn-mediated biologics-transcytosis may be influenced by environmental factors. Röhm et al. used a nebulization platform to evaluate different formulations of therapeutics containing biologics, and five different excipients (i.e., arginine, cyclodextrin, polysorbate, sorbitol, and trehalose) with three concentration levels were tested for each. Three different formulations (F1, F2, and F3) containing different concentrations of trehalose, sorbitol, arginine, polysorbate and cyclodextrin were tested to minimize aerosolization-induced protein aggregation and maximize permeation through an in vitro epithelial cell barrier [35]. After a 90 min or 240 min incubation time, protein concentrations were determined in the abluminal media by either ELISA or fluorescence spectroscopy. F1 reduced the aggregation of native Fab and IgG relative to vehicle by up to 50% and enhanced the transepithelial permeation rate up to 2.8-fold in comparison to the vehicle. F2 also improved the transport rate, but F3 did not [35].

In 2019, Ladel et al. evaluated the influence of IgG glycosylation on transcytosis by comparing the permeation rate of deglycosylated (DG) hIgGs to that of wild-type (WT) hIgGs. They demonstrated that the permeation rate was significantly higher for DG hIgG than for WT hIgG in both OEPC and RPMI cells. Interestingly, the permeation rate and flux through OEPC cells were significantly higher than the permeation rate through RPMI cells for both DG hIgGs and hIgGs, thus suggesting that transcytosis efficiency also depends on cell type. Finally, species-dependent binding to IgG receptors or species-dependent IgG trafficking and/or degradation pathways influenced the permeation of IgGs through the olfactory mucosa, and it was shown that hIgGs reached higher levels than pIgGs. The authors concluded that the permeation behaviour of DG and WT hIgG displayed similar patterns in in vitro and ex vivo models [16].

### 3.5. Toxicity of Intranasal Delivery

Only three out of the 12 studies included evaluated the potential toxicity of biologics’ intranasal delivery. Globally, all of these studies found that the intranasal delivery of biologics was generally safe and did not induce specific side effects. Bequignon et al. assessed the toxicity of infliximab intranasal delivery using two methods. Transepithelial electrical resistance measurements were used to assess monolayer permeability, and trypan blue exclusion was used to assess cellular viability. It is important to note that no cellular toxicity was observed using either method after a 4 h incubation period at 37 °C with infliximab [9]. In addition, Samson et al. performed histological analyses after intranasal delivery of bevacizumab. There was no evidence of histological effects, confirming that nasal delivery of bevacizumab was harmless [46].

### 3.6. Selected Studies Drawbacks

Drawing strong conclusions about intranasal delivery of biologics may be premature owing to the small number of studies included and the heterogeneity in the models used, as well as biologics tested and the reported results.

The doses of biologics tested varied a lot among the studies, even for similar study designs: (i) for in vivo studies from 1 µg [69] to 2.5 mg [61], considering 10 to 20 g per mouse; (ii) for in vitro studies from 12.5 ng [9] to 4 mg [35]; and (iii) for ex vivo studies from 8 µg [15] to 500 µg [46]. None of the studies tried to identify a saturation kinetic.

The time for collecting data varied widely across studies. In vivo assessment of the efficiency and/or toxicity of biologics generally used a 1 month follow-up in the majority of the studies, but some had a follow-up of over 6 months after intranasal delivery [29]. The time of incubation used in the in vitro and ex vivo studies ranged from 30 min [61] to 6 days [15,16,26,46]. During these periods, samplings were performed regularly.

The nasal epithelial cells used for transcytosis assays of biologics were either primary cultures of HNECs [9,10], human-derived RPMI cells [16,35] or porcine-derived OEPCs [16]. In vivo studies were performed on mice in five studies [26,27,28,29,69] and on rats in one study [61]. Both ex vivo studies were performed on porcine olfactory mucosae [15,16]. Regarding animal studies, both BALB/c [26,27] and C57BL/6 [27,28,29,69] backgrounds were used as WT; these were mostly 6- to 8-week-old females [28,29] but also 8- to 12-week-old females [69]. FcRn knockout (KO) mice on a C57BL/6 background [28,29] and on a BALB/c background [26] were also used. Bern et al. also specifically studied a human FcRn transgenic mouse model that lacked expression of both mouse FcRn and mouse albumin [B6.Cg-Albem12 Mvw Fcgrttm1Dcr Tg(FCGRT)32Dcr/MvwJ; homozygous Tg32 alb knockout (KO) mice] [26]. Finally, Kumar et al. purchased adult female Sprague Dawley rats (180–200 g; Envigo) for all in vivo experiments. Some studies used both in vitro and in vivo models to study transcytosis and interactions with FcRn, but the cells used in vitro were not airway epithelial nasal cells; thus, their results are not described in this review [26,28,29].

## 4. Discussion

This systematic review focused on the role of FcRn in the nasal administration of biologics. For almost twenty years, researchers have demonstrated the involvement of FcRn in IgG transport in various tissues and cell types [7,11,32,70,71]. Accordingly, all of the studies included in this review demonstrated efficient transcytosis [9,15,16,26,27,28,29,35,46,61,69] of all of the biologics tested, irrespective of their molecular weight or the dose administered; therefore, they indicate that nasal administration may represent an efficient administration route for biologics for inducing both local and systemic effects. However, several limitations in the literature were identified. First, the doses used to obtain efficient transport through the nasal or olfactory epithelium differed dramatically among the studies, and no meta-analysis could be conducted. Second, regarding the well-known pharmacokinetic characteristics, pH plays a huge role; yet, it was not specified in most of the included studies [10,15,27,28,29,35,46,61,69]. Third, the type of sample used was heterogeneous, mostly because of the design of the studies. All but one in vivo study used mice as a model [26,27,28,29,69], all but one ex vivo study used porcine olfactory mucosa [15,16,46] and the in vitro studies were either on HNECs [9], RPMI cells [16,35] or OEPC [16]. The nasal route is a promising non-invasive method of delivery for mAbs to treat respiratory diseases, as it has demonstrated therapeutic responses in various models and leads to high mAb concentrations in the lungs, while limiting mAb passage into systemic circulation [56,72,73,74].

### 4.1. FcRn Expression

Among the 12 studies included in this systematic review, six first confirmed the expression of FcRn in their different models that were chosen to assess biologic-transcytosis across airway epithelial cells [9,10,15,16,28,29]. However, they did not all demonstrate where FcRn was expressed, and the exact mechanism underlying transcytosis is unclear. Some authors have demonstrated that FcRn is preferentially expressed at the basolateral side of cells in humans [10]. Others used immunofluorescence staining against FcRn in OEPC, and showed that FcRn is not expressed in all epithelial cells but varies heavily in the cellular monolayer, indicating that FcRn expression is highly dependent on the cell type [75]. This finding supports the common assumption that uptake is based mainly on pinocytosis, reinforcing the idea that nasal epithelial IgG trafficking is only FcRn-dependent. Such an assumption would suppose that FcRn-dependent IgG transport is species-independent [76]. In contrast, other authors excluded the notion that nasal epithelial IgG trafficking is only FcRn-dependent because of the insensitivity of FcRn to Fc-deglycosylation [16,77,78]. Access to the CNS after intranasal administration of biologics is also believed to involve both FcRn-dependent mechanisms and an FcRn-independent mechanism. The intranasal delivery of macromolecules may allow them to be absorbed into the systemic circulation via nasal blood vessels and access the CNS by crossing the blood–brain barrier/blood–cerebrospinal fluid barriers, or they may access the CNS via direct perineural, perivascular and lymphatic pathways that exist in the nasal mucosa [44,75,77,79,80].

### 4.2. Transcytosis Models

In general, the amount of deposited drug and the percentage of transported drug are not comparable from one study to another, as they differ dramatically when altering experimental conditions. Moreover, different types of samples were used to test the nasal route, which made it difficult to compare transcytosis efficiency from one study to another. Despite this heterogeneity, the nasal epithelial cell line RPMI used by Röhm [35] and Ladel [16] is well characterized in the literature and was used in several studies as a model for the respiratory epithelium of the nasal cavity and the bronchus [81,82]. Indeed, this cell line, derived from cancerous human nasal squamous epithelium, is currently used as a standard model of intranasal drug delivery, since specimens of human nasal mucosa are generally unavailable [83,84]. In addition, the primary culture of HNECs used by Bequignon et al. [9] has previously been well-demonstrated in several experiments [85,86]. The in vitro model reported in this review is well characterized. Nevertheless, another limitation regarding the lack of correlation between FcRn binding characteristics in vitro and in vivo could be raised. Indeed, several factors may contribute to this lack of a correlation: (i) for biologics that bind negligibly to FcRn at near neutral pH, uptake of mAbs into cells is dependent on fluid phase pinocytosis [87]; (ii) for some biologics, the endosomal environment (salt concentration, temperature, etc.) may result in different binding properties than those observed when carrying out interaction analyses in vitro, typically at 25 °C; and (iii) degradation or modification of biologics during storage can lead to a loss of binding affinity for FcRn [44]. Given the variability in binding constants from one laboratory to another, it may also be instructive to develop a standardized protocol for FcRn–IgG interaction studies, as discussed in other studies [88,89,90].

Another possibility is to use ex vivo models. Porcine mucosa has been used to model the human nasal mucosa. This is interesting because the mucosa of both porcine and humans are highly similar, and the cross-species transport of human IgG by porcine FcRn has already been shown by Stirling et al. [15,76,83,84,91]. In the ex vivo model, more complex factors interfere with IgG permeation, such as the interaction with the local immune system and uptake in neuronal fibres, which make it more similar to in vivo conditions [16].

Finally, in vivo models can be used, such as mouse models. Variations in residue encompassing the FcRn–IgG interaction site across species lead to differences in binding behaviour that have direct relevance to the use of mice as preclinical models [41,92,93]. Unfortunately, mice also possess a higher affinity for FcRn, as well as a greater capacity to form clearance-enhancing anti-drug antibodies. In addition, mice have lower levels of endogenous IgGs, which results in a lower level of FcRn binding competition than in humans [94]. Collectively, these characteristics undermine the relevance of WT mice as models for human Ab pharmacokinetics [95,96]. Moreover, species-related differences must be considered. For example, unlike in humans, porcine maternal IgGs do not cross the porcine placenta to reach the foetus, even though FcRn is expressed there [97]. Unlike FcRn in mice, monkey FcRn binds to human albumin with similar binding kinetics as albumin from the same species [98]. Thus, nonhuman primates are likely to be a good model to assess the effect of biotinylated albumin on the delivery and plasma half-life of human albumin fusions before testing in humans [99]. Nonhuman primates, especially cynomolgus monkeys, are also the models of choice to probe FcRn dynamics due to their relatively robust ability to recapitulate human-like pharmacokinetic properties [95,100]. However, high-throughput screens in cynomolgus monkeys are impractical due to financial and ethical constraints. Mice, on the other hand, can easily be used with significantly higher throughput. Several transgenic mouse models have been developed in the absence of high-throughput preclinical models with human-like FcRn dynamics that express human FcRn to varying degrees [101,102,103,104,105].

### 4.3. Factors Influencing Nasal Transcytosis

It is well known that the FcRn/IgG interaction is highly dependent on pH. FcRn binds IgG with nanomolar affinities at a pH of 6.5–6.0, which is found in intracellular vesicles, and shows negligible interactions at a pH of 7.4–7.0 [92], which allows for IgG release in the bloodstream [93]. The pH of the mucous layer in the nose is reported to be approximately 6, which is another argument favouring the use of the nasal route [38]. However, the influence of apical pH on transcytosis efficiency was not assessed in nine out of the 12 studies included in this review [10,15,27,28,29,35,46,61,69]. The pH used for the experiments was only reported in one [16] of the four ex vivo studies included [10,15,16,46], but the authors did not discuss the impact it may have had on the results. In contrast, Bequignon et al. clearly demonstrated that an acidic pH (pH = 6) was necessary for efficient transcytosis [9]. Finally, only one out of the six in vivo studies [26,27,28,29,61,69] included reported a pH for intranasal administration of scFv-Alb (pH = 5.5) [26]. IgG transcytosis through the nasal mucosa seemed relatively rapid, with mAbs detected 4 h after incubation with airway-derived cells [9]. In addition, IgG transcytosis has been shown to be dose-dependent [9,26].

Subsequent work demonstrated that intranasally applied low molecular weight peptides, e.g., oxytocin (1 kDa), readily access the CSF of rodents [71,92], monkeys [93] and humans [81]. In addition, the nasal route for biologics administration is not always successful. The most important limiting factors for the nasal absorption of high molecular weight molecules such as mAbs are low epithelial membrane permeability and mucociliary clearance [106]. Both factors are included in the cellular OEPC model used by Ladel et al. [16].

The degradation or modification of biologics during aerosolization can lead to a loss of binding affinity for FcRn. Proteins are sensitive to aerosolization-associated shear stress, and may lose biological activity if used in aerosols [107,108]. Storage conditions may also impact the binding affinity of biologics for FcRn. It has been shown that oxidation of one methionine residue, Met252, in proximity to the human FcRn–IgG1 interaction site can lead to a decreased in vivo half-life [79,80,109,110].

Another factor that may result in different binding properties is temperature [44], but little data were available in the included studies.

### 4.4. Biologics Integrity

Questions regarding the integrity of transcytosed biologics are rising, as evidence of the efficiency of nasal administration gradually accumulates. SDS-PAGE and autoradiographic analysis of the brain soluble protein fraction yielded distinct heavy and light chain [^125^I]-labelled protein bands following intranasal administration of radiolabelled [^125^I]-IgG in the study of Kumar et al. in 2018 [61]. Such detection of heavy and light chain bands has generally been considered consistent with intact IgG being present in vivo, e.g., in studies analysing the endogenous and exogenous IgG content in the brain [111]. The efficacy of immunization in a mouse model challenged with Ft, HSV-2 or HIV is also proof of biological integrity [27,28,29,69]. Indeed, the mice survived an intravaginal challenge weeks after intranasal administration of ICs. Intranasal administration is efficient, and biologics maintain their integrity after reaching systemic circulation. After acknowledging this, it is still unclear as to how much went through and the exact pathway used. Kumar et al. demonstrated significantly higher [^125^I]-IgG concentrations within the CNS after intranasal administration compared to intra-arterial doses that produced similar end-point blood [^125^I]-IgG levels. Nevertheless, these concentrations were still low and indicate that only a small fraction of intranasally administered [^125^I]-IgG accessed the CNS at an early time point (30 min) following a single acute dose [61]. Repeated intranasal dosing with smaller doses may be considered to achieve similar or higher concentrations [61].

Another possibility to enhance transepithelial transport is the administration of tight junction modulators. This has been shown to improve macromolecule transport across numerous epithelial barriers throughout the body, including at nasal sites [112]. Indeed, permeation enhancement at the nasal epithelium is expected to yield higher drug and tracer levels in the lamina propria, where nerve-associated pathways to the brain may be most accessible [60]. Matrix metalloproteinase-9 (MMP-9), a type IV collagenase and member of a large class of zinc-dependent endopeptidases, has been shown to be involved in extracellular matrix (ECM) remodelling [113], as well as tight junction modulation, in part through alteration of claudin-1 [114]. MMP-9 is naturally expressed in its active form in the olfactory mucosa, where it likely plays a role in the continual replacement of epithelial cells (re-epithelialization) and olfactory sensory neurons during regular cell turnover [115]. Recently, it has been shown that intranasal MMP-9 can be used as a local nasal permeability enhancer [116]. It is important to keep in mind that nasal permeability enhancers cause significant nasal irritation and mucosal toxicity [117], and additional studies are needed.

Although most therapeutic mAbs are delivered intravenously, several are delivered via other routes. The subcutaneous route offers the major advantage that mAbs can be self-administered, prompting interest in understanding the processes through which subcutaneously delivered therapeutics, including mAbs, enter circulation [45]. Knowledge of these processes may inform antibody engineering strategies to increase the effective therapeutic dose.

## 5. Conclusions and Future Perspectives

This systematic review confirmed the expression of FcRn in nasal airway and olfactory epithelium, as well as its potential role in IgG transcytosis across airway-polarized cell layers. It is now clear that FcRn enables the bi-directional transfer of IgG molecules, i.e., to deliver IgG into the lumen from the tissue space, as well as the return of IgG-bound luminal antigens back into the lamina propria. Choosing intranasal delivery over oral delivery for biologics may be a great non-invasive method to induce systemic effects, as it would avoid biologics’ degradation by the extremely low pH and the presence of digestive enzymes in the gastrointestinal tract [66]. In addition, intranasal delivery may represent a comfortable and non- to minimally invasive way of self-administration [36].

Major progress has been made with regard to FcRn-mediated mucosa-transcytosis. However, further developments are required for better understanding the pharmacokinetic and pharmacodynamics properties of FcRn-mediated IgG-transcytosis through mucosal tissue. Specific studies evaluating the dose–response effect, the timing of FcRn-mediated igG-transcytosis and the uptake processes in an inflammatory environment should be initiated. Further, variations in these characteristics across cell types, such as olfactory cells vs. ciliated cells, are unexplored. Whether IgG-transcytosis is exclusively dependent on FcRn should also be further studied using an FcRn antagonist. Altogether, the characterization of intranasal delivery of biologics and the role of FcRn in this setting are expected to increase in the near future. This would be helpful for the clinical development of the intranasal delivery of various biologics, including mAbs or modified Fc-fusion proteins, for the treatment of a wide range of diseases.

## Figures and Tables

**Table 1:** Characteristics of the included studies.

First Author Date	Design	Species	Intervention	Application	Biotherapy Tested	Dose Administered	Concentration	Formulation	Primary Outcome	Blocking FcRn
Rawool [27] * 2008, India	In vivo	Mice FcRn WT	Assess FcRn-mediated mucosal vaccine delivery with an Ft model	Vaccine delivery against Ft	mAb-iFT (fusion of iFT and IgG)	2 × 10^7^ iFT/mAB-iFT/F(ab′)2-iFT organisms	2 × 10^7^ CFU/20 µL	PBS	FcRn-targeted immunogen enhanced immunogen-specific IgA production and protection against subsequent infection. It is a highly effective vaccination strategy against Ft.	Yes
Lu [28] * 2011, USA	In vivo	Mice FcRn WT and KO	Assess ability of FcRn to deliver Gag-Fc fusion protein in a HIV model	Vaccine delivery against VIH	HIV Gag-Fc fusion protein	20 µg	1 mg/mL	PBS	FcRn-targeted mucosal immunization was effective at inducing Gag specific Ab responses in serum or mucosal secretions, and high levels of stable immune memory were obtained.	Yes
Ye [29] * 2011, USA	In vivo	Mice FcRn WT and KO	Assess FcRn-mediated mucosal vaccine delivery with an HSV-2 model	Vaccine delivery against HSV	gD-Fc/wt (HSV-2 gD fused with an IgG Fc fragment)	20 µg	1 mg/mL	PBS	Intranasal immunization with an engineered fused protein resulted in complete protection of wild-type, but not FcRn KO, mice that were intravaginally challenged with virulent HSV-2.	Yes
Bitsatksis [69] * 2015, USA	In vivo	Mice FcRn WT	Assess FcRn-mediated mucosal vaccine delivery with an Ft model	Vaccine delivery against Ft	mAb-iFT IC (fusion of iFT and IgG)	2 × 10^7^ CFU mAb-iFT IC organisms	NA	PNS	FcRn targeting increases the frequency and activation status of DCs in the lungs of immunized mice and mediates the generation of Ft-specific effector memory CD4^+^ T cells.	No
Kumar [61] * 2018, USA	In vivo	Rats FcRn WT	Assess CNS IgG distribution after intranasal administration	Intracerebral mAb delivery	Radiolabeled Ab: [^125^I]-IgG	50 µg to 2.5 mg for [^125^I]-IgG	1-20-50 mg/mL	PBS	[^125^I]-IgG concentrations in the CNS was higher following intranasal delivery compared to intra-arterial delivery for doses producing similar endpoint blood concentrations.	No
					Fluorescently labeled Ab: AF488-IgG	0.7 mg for AF488-IgG	30 mg/mL			
Bern [26] * 2020, Norway	In vivo	Mice FcRn WT and KO hFcRn	Assess ability of FcRn for delivery of albumin-based biologics	Haemophilia	Biotinylated albumin (WT, KAHQ or QMP) IgG1 and scFv-Alb	10 to 30 µg for a 10 g mice	20 µL à la dose de 1 mg/kg to 3.2 mg/kg	PBS	Nasal FcRn enabled efficient transcytosis of albumin fusion proteins.	Yes
Röhm [35] * 2017, Germany	In vitro	RPMI cells	Assess IgG quality after transcytosis	Enhancement of IgG permeation rate	mAb HIRMab 83-14	4 mg	1.4 mg/mL for Fab; 4 mg/mL for IgG	L-arginine, HBC, PS20, sorbitol, trehalose	Aggregation of native IgG was reduced and transepithelial permeation rate was enhanced up to 2.8-fold with the used of specific formulations (F1) for intranasal aerosol-cell delivery.	No
Bequignon [9] * 2019, France	In vitro	HNEC	Assess mAb transcytosis via FcRn	Anti-cancer immunotherapy	Infliximab	12.5 to 1250 ng	50-500-50000 µg/mL	HBSS-MES	Transepithelial passage of therapeutic mAb was dose-dependent.	No
Ladel [16] * 2019, Germany	In vitro	OEPC and RPMI cells	Assess permeation rates of IgGs through the nasal mucosae	Anti-cancer immunotherapy	WT pIgG, WT hIgG and DG hIgG (biosimilar of Bevacizumab)	50 µg	1.5 mg/mL	PBS	hIgG permeation was faster than pIgGs over the first four in OEPC ALI cultures, but it converges from 8 h to 48 h. DG hIgG showed a higher permeation rate than WT hIgG in the RPMI ALI model.	No
	Ex vivo	Porcine olfactory mucosa							hIgG permeation was 12 times higher after 5 h than the one of pIgG. The permeation rate of DG hIgG and WT hIgG did not show differences.	
Samson [46] * 2012, France	Ex vivo	Porcine nasal mucosa	Assess the transport of bevacizumab through porcine nasal mucosa	Rendu-Osler Disease	Bevacizumab	500 µg	25 mg/mL	trehalose, sodium phosphate, PS20, water	Total recovery of intranasally-delivered bevacizumab was 83% of the initial dose, with 53% localized at the mucosal surface and 11% that had gone through the mucosa.	No
Heidl [10] * 2015, Austria	Ex vivo	HNEC	Assess FcRn expression and localization in HNEC	Intranasal administration of mAb	None	NA	NA	NA	FcRn was detected in ciliated and basal cells of the nasal epithelium as well as in vascular endothelial cells and in gland tissue.	No
Ladel [15] * 2018, Germany	Ex vivo	Porcine olfactory mucosa	Assess ability of FcRn to transport IgGs through the nasal lamina propria	Anti-cancer immunotherapy	pIgG and hIgG (biosimilar of Bevacizumab)	8 µg	8 mg/mL	PBS	FcRn is expressed in the olfactory mucosa and enabled the apical uptake of allogeni, and xenogenic IgG in a species-specific manner.	No

* See Supplementary File S2 for a complete report of the quality evaluation of included studies according to the OHAT for assessing risk of bias. Abbreviations: CFU, Colony Forming Unit; CNS, Central Nervous system; DC, Dendritic Cells; DG, deglosylated; FcRn, neonatal Fc Receptor; FVII, Factor VII; Ft, Francisella Tularensis; gD, glycoprotein D; hFcRn, human FcRn; HBC, 2-Hydroxypropyl-β-cyclodextrin; hIgG, human IgG; HIV Gag-Fc, fusion of p24 protein from HIV Gag with IgG heavy chain; HNEC, Human Nasal Epithelial Cell; iFt, inactivated Francisella tularensis; KO, knock-out; mAb, Monoclonal Antibody; NA, not available; PS20, Polysorbate 20; PBS, Phosphate Buffer Saline; pIgG, porcine IgG; QMP, triple mutant E505Q/T527M/K573P Albumin engineered; rFVII, recombinant Factor VII; RPMI cells, carcinoma from squamous epithelium obtained from a human nasal septum; scFv-Alb, single-chain variable fragment fused to Albumin; WT, wild-type.

**Table 2:** Risk of bias summary using OHAT.

Study	Selection Bias	Performance Bias	Attrition Bias	Detection Bias	Selective Reporting	Other Bias
Rawool [27], 2008	--	--	+	+	+	+
Lu [28], 2011	--	--	+	+	++	+
Ye [29], 2011	--	--	++	++	++	+
Bitsatksis [69] 2015	--	--	++	+	++	+
Kumar [61] 2018	--	--	+	+	+	-
Bern [26] 2020	--	--	++	+	++	+
Röhm [35] 2017	--	--	++	+	++	+
Bequignon [9] 2019	--	--	++	+	++	+
Samson [46] 2012	--	--	++	++	++	+
Heidl [10] 2015	--	--	+	-	+	+
Ladel [15] 2018	--	--	+	+	++	+
Ladel [16] 2019	--	--	+	+	++	+

(--) Definitely high risk; (-) probably high risk; (+) probably low risk; (++) definitely low risk.

## Data Availability

Not Applicable.

## Supplementary Materials

The following are available online at https://www.mdpi.com/article/10.3390/ijms22126475/s1, Supplementary File S1: Detailed Search Strategy and Equations Used, Supplementary File S2: Detailed Risk of Bias Using OHAT, Supplementary File S3: Type of Samples and Protocols of the Included In Vivo Studies, Supplementary File S4: Type of Samples and Protocols of the Included In Vitro and Ex Vivo Studies.
