# Supplementary material for: FcRn as a Transporter for Nasal Delivery of Biologics: A Systematic Review
Source: Int J Mol Sci. 2021 Jun 17;22(12):6475. doi: 10.3390/ijms22126475 (PMC8234196; doi:10.3390/ijms22126475)
Supplement: Supplementary file 1 [file ijms-22-06475-s001.zip › supplementary/Supplementary File S1.pdf]

Supplementary File S1. Detailed Search Strategy and Equations Used

**1/ Pubmed [including Medline] : 468 results**
(("Fc receptor, neonatal"[Supplementary Concept] OR "Receptors, Fc"[Mesh:NoExp] OR "Receptors, IgG"[Mesh]) AND ("Nose"[Mesh] OR "Respiratory System"[Mesh:NoExp] OR "Respiratory Mucosa"[Mesh:NoExp] OR "Nasal Mucosa"[Mesh] OR "Mucous Membrane"[Mesh:NoExp] OR "Epithelial Cells"[Mesh:NoExp] OR "Alveolar Epithelial Cells"[Mesh] OR ("Dendritic Cells"[Mesh] AND ("Nose"[Mesh] OR "Respiratory System"[Mesh:NoExp] OR "Respiratory Mucosa"[Mesh:NoExp] OR "Nasal Mucosa"[Mesh] OR "Mucous Membrane"[Mesh:NoExp])) OR "Goblet Cells"[Mesh] OR "Neuroepithelial Cells" [Mesh] OR "Epithelium"[Mesh:NoExp] OR "Basement Membrane"[Mesh:NoExp] OR "Basilar Membrane"[Mesh] OR "Administration, Intranasal"[Mesh] OR "Administration, Mucosal"[Mesh:NoExp] OR "Administration, Topical"[Mesh:NoExp] OR "Administration, Inhalation"[Mesh] OR "Lung"[Mesh]) AND ("Aerosols"[Mesh] OR "Drug Delivery Systems"[Mesh] OR "Drug Evaluation, Preclinical"[Mesh] OR "Nebulizers and Vaporizers"[Mesh] OR "Dose-Response Relationship, Drug"[Mesh] OR "Molecular Targeted Therapy"[Mesh] OR "Biological Transport"[Mesh] OR "Recombinant Fusion Proteins"[Mesh] OR "Protein Engineering"[Mesh] OR "Antibodies, Monoclonal"[Mesh] OR "Antibody Affinity"[Mesh] OR "Autoimmune Diseases"[Mesh] OR "Binding Sites, Antibody"[Mesh] OR "Half-Life"[Mesh] OR "Histocompatibility Antigens Class I"[Mesh] OR "Models, Molecular"[Mesh] OR "Biological Availability"[Mesh] OR "Permeability"[Mesh] OR "Endocytosis"[Mesh] OR "Transcytosis"[Mesh] OR "Receptors, Albumin"[Mesh] OR "Albumins"[Mesh:NoExp] OR "Immunoglobulin Fc Fragments"[Mesh] OR "Immunoglobulin G"[Mesh] OR "Immunity, Mucosal"[Mesh] OR "Respiratory System Agents"[Pharmacological Action] OR "Pharmacokinetics"[Mesh] OR "pharmacokinetics"[Subheading] OR "Homeostasis"[Mesh] OR "Ligands"[Mesh] OR "adverse effects"[Subheading] OR "toxicity"[Subheading]) AND (English[lang] OR French[lang]))
OR
((FcRn[tiab] OR (Fc[tiab] AND Recept*[tiab] AND neonat*[tiab]) OR (Fcgrt[tiab] AND protein*[tiab])) AND (Nose*[tiab] OR Nasal*[tiab] OR Intranasal*[tiab] OR Mucosal*[tiab] OR Mucous*[tiab] OR Epitheli*[tiab] OR Neuroepitheli*[tiab] OR ((Dendritic*[tiab] OR Goblet*[tiab]) AND Cell*[tiab]) OR ((Basement*[tiab] OR Basal*[tiab] OR Basilar*[tiab]) AND (Membrane*[tiab] OR Lamina*[tiab])) OR Inhal*[tiab] OR Inspirat*[tiab] OR Lung*[tiab]) AND (Aerosol*[tiab] OR "Drug Delivery Systems"[tiab] OR Nebuliz*[tiab] OR Vaporiz*[tiab] OR "Molecular Targeted Therap*"[tiab] OR "Biological Transport*"[tiab] OR "Recombinant Fusion Protein*"[tiab] OR (Protein*[tiab] AND Engineering*[tiab]) OR ((Antibod*[tiab]) AND (monoclonal[tiab] OR affinit*[tiab] OR "binding site*"[tiab])) OR "Autoimmune Disease*"[tiab] OR "Half-Life*"[tiab] OR Halflife*[tiab] OR (Histocompatibilit*[tiab] AND Antigen*[tiab] AND "Class I"[tiab]) OR (Model[tiab] AND Molecular*[tiab]) OR ((Biologic*[tiab] OR Physiologi*[tiab] OR Equivalen*[tiab]) AND Availabilit*[tiab]) OR Permeabilit*[tiab] OR Endocytos*[tiab] OR Transcyto*[tiab] OR ((Transcyto*[tiab] OR Transcellular*[tiab]) AND (Pathway*[tiab] OR Transport*[tiab])) OR (Receptor*[tiab] AND Albumin*[tiab]) OR "Immunoglobulin G"[tiab] OR (Immun*[tiab] AND Mucosal[tiab]) OR "Respiratory System Agents"[tiab] OR Pharmacokinetic*[tiab] OR Homeostas*[tiab] OR Ligand*[tiab] OR "adverse effect*"[tiab] OR toxicit*[tiab]) AND (English[lang] OR French[lang]))

**2/ Web of Science [Clarivate Analytics] : 413 results**
(from Web of Science Core Collection)
You searched for: TOPIC: ((FcRn OR (Fc AND Recept* AND neonat*) OR (Fcgrt AND protein*))) AND TOPIC: ((Nose* OR Nasal* OR Intranasal* OR Mucosal* OR Mucous* OR Epitheli* OR Neuroepitheli* OR ((Dendriti* OR Goblet*) AND Cell*) OR ((Basement* OR Basal* OR Basilar*) AND (Membrane* OR Lamina*)) OR Inhal* OR Inspirat* OR Lung* OR nez OR muqueu* OR (cellule* AND (gobelet* OR caliciforme*)) OR (membrane* AND basilaire*) OR poumon*)) AND TOPIC: ((Aerosol* OR "Drug Delivery Systems" OR Nebuli* OR Vapori* OR "Molecular Targeted Therap*" OR "Biological Transport*" OR "Recombinant Fusion Protein*" OR (Protein* AND Engineering*) OR ((Antibod*) AND (monoclonal OR affinit* OR "binding site*")) OR "Autoimmune Disease*" OR "Half-Life*" OR Halflife* OR (Histocompatibilit* AND Antigen* AND "Class I") OR (Model AND Molecular*) OR ((Biologic* OR Physiologi* OR Equivalen*) AND Availabilit*) OR Permeabilit* OR Endocytos* OR Transcyto* OR ((Transcyto* OR Transcellular*) AND (Pathway* OR Transport*)) OR (Receptor* AND Albumin*) OR "Immunoglobulin G" OR (Immun* AND Mucosal) OR "Respiratory System Agents" OR Pharmacokinetic* OR Homeostas* OR Ligand* OR "adverse effect*" OR toxicit* OR medicament* OR "maladie* autoimmun*" OR "maladie* auto-immun*" OR pharmacociné* OR ((Effet* OR événement* OR incident* OR réaction*) AND (indésirable* OR secondaire* OR délétère* OR néfaste* OR noci* OR nuisible*)) OR "thérapie* moléculaire* ciblée*" OR "protéine* de fusion* recombinante*" OR ((affinité* OR monoclona* OR site* OR fix*) AND anticorp*) OR Paratope* OR "Demi-vie" OR "demi vie" OR "modèle* moléculaire*" OR biodisponibilité* OR bio-disponibilité*)) AND LANGUAGE: (English OR French)
Timespan: 1900-2021. Indexes: SCI-EXPANDED, SSCI, A&HCI, CPCI-S, CPCI-SSH, ESCI, CCR-EXPANDED, IC.
**3/ Scopus [Elsevier] : 339 results**
(TITLE-ABS-KEY ((fcrn OR (fc AND recept* AND neonat*) OR (fcgrt AND protein*))) AND TITLE-ABS-KEY ((nose* OR nasal* OR intranasal* OR mucosal* OR mucous* OR epitheli* OR neuroepitheli* OR ((dendriti* OR goblet*) AND cell*) OR ((basement* OR basal* OR basilar*) AND (membrane* OR lamina*)) OR inhal* OR inspirat* OR lung* OR nez OR muqueu* OR (cellule* AND (gobelet* OR caliciforme*)) OR (membrane* AND basilaire*) OR poumon*)) AND TITLE-ABS-KEY ((aerosol* OR "Drug Delivery Systems" OR nebuli* OR vapori* OR "Molecular Targeted Therap*" OR "Biological Transport*" OR "Recombinant Fusion Protein*" OR (protein* AND engineering*) OR ((antibod*) AND (monoclonal OR affinit* OR "binding site*")) OR "Autoimmune Disease*" OR "Half-Life*" OR halflife* OR (histocompatibilit* AND antigen* AND "Class I") OR (model AND molecular*) OR ((biologic* OR physiologi* OR equivalen*) AND availabilit*) OR permeabilit* OR endocytos* OR transcyto* OR ((transcyto* OR transcellular*) AND (pathway* OR transport*)) OR (receptor* AND albumin*) OR "Immunoglobulin G" OR (immun* AND mucosal) OR "Respiratory System Agents" OR pharmacokinetic* OR homeostas* OR ligand* OR "adverse effect*" OR toxicit* OR medicament* OR "maladie* autoimmun*" OR "maladie* auto-immun*" OR pharmacociné* OR ((effet* OR événement* OR incident* OR réaction*) AND (indésirable* OR secondaire* OR délétère* OR néfaste* OR noci* OR nuisible*)) OR "thérapie* moléculaire* ciblée*" OR "protéine* de fusion* recombinante*" OR ((affinité* OR monoclona* OR site* OR fix*) AND anticorp*) OR paratope* OR "Demi-vie" OR "demi vie" OR "modèle* moléculaire*" OR biodisponibilité* OR bio-disponibilité*)) AND LANGUAGE (english OR french))

**4/ Cochrane Library [Wiley] : 3 results (1 Review + 2 Trials)**
1 Cochrane Review matching (FcRn OR (Fc AND Recept* AND neonat*) OR (Fcgrt AND protein*)) in Title Abstract Keyword AND (Nose* OR Nasal* OR Intranasal* OR Mucosal* OR Mucous* OR Epitheli* OR Neuroepitheli* OR ((Dendriti* OR Goblet*) AND Cell*) OR ((Basement* OR Basal* OR Basilar*) AND (Membrane* OR Lamina*)) OR Inhal* OR Inspirat* OR Lung* OR nez OR muqueu* OR (cellule* AND (gobelet* OR caliciforme*)) OR (membrane* AND basilaire*) OR poumon*) in Title Abstract Keyword AND (Aerosol* OR "Drug Delivery Systems" OR Nebuli* OR Vapori* OR "Molecular Targeted Therap*" OR "Biological Transport*" OR "Recombinant Fusion Protein*" OR (Protein* AND Engineering*) OR ((Antibod*) AND (monoclonal OR affinit* OR "binding site*")) OR "Autoimmune Disease*" OR "Half-Life*" OR Halflife* OR (Histocompatibilit* AND Antigen* AND "Class I") OR (Model AND Molecular*) OR ((Biologic* OR Physiologi* OR Equivalen*) AND Availabilit*) OR Permeabilit* OR Endocytos* OR Transcyto* OR ((Transcyto* OR Transcellular*) AND (Pathway* OR Transport*)) OR (Receptor* AND Albumin*) OR "Immunoglobulin G" OR (Immun* AND Mucosal) OR "Respiratory System Agents" OR Pharmacokinetic* OR Homeostas* OR Ligand* OR "adverse effect*" OR toxicit* OR medicament* OR "maladie* autoimmun*" OR "maladie* auto-immun*" OR pharmacociné* OR ((Effet* OR événement* OR incident* OR réaction*) AND (indésirable* OR secondaire* OR délétère* OR néfaste* OR noci* OR nuisible*)) OR "thérapie* moléculaire* ciblée*" OR "protéine* de fusion* recombinante*" OR ((affinité* OR monoclona* OR site* OR fix*) AND anticorp*) OR Paratope* OR "Demi-vie" OR "demi vie" OR "modèle* moléculaire*" OR biodisponibilité* OR bio-disponibilité*) in Title Abstract Keyword - (Word variations have been searched)
2 Trials matching (FcRn OR (Fc AND Recept* AND neonat*) OR (Fcgrt AND protein*)) in Title Abstract Keyword AND (Nose* OR Nasal* OR Intranasal* OR Mucosal* OR Mucous* OR Epitheli* OR Neuroepitheli* OR ((Dendriti* OR Goblet*) AND Cell*) OR ((Basement* OR Basal* OR Basilar*) AND (Membrane* OR Lamina*)) OR Inhal* OR Inspirat* OR Lung* OR nez OR muqueu* OR (cellule* AND (gobelet* OR caliciforme*)) OR (membrane* AND basilaire*) OR poumon*) in Title Abstract Keyword AND (Aerosol* OR "Drug Delivery Systems" OR Nebuli* OR Vapori* OR "Molecular Targeted Therap*" OR "Biological Transport*" OR "Recombinant Fusion Protein*" OR (Protein* AND Engineering*) OR ((Antibod*) AND (monoclonal OR affinit* OR "binding site*")) OR "Autoimmune Disease*" OR "Half-Life*" OR Halflife* OR (Histocompatibilit* AND Antigen* AND "Class I") OR (Model AND Molecular*) OR ((Biologic* OR Physiologi* OR Equivalen*) AND Availabilit*) OR Permeabilit* OR Endocytos* OR Transcyto* OR ((Transcyto* OR Transcellular*) AND (Pathway* OR Transport*)) OR (Receptor* AND Albumin*) OR "Immunoglobulin G" OR (Immun* AND Mucosal) OR "Respiratory System Agents" OR Pharmacokinetic* OR Homeostas* OR Ligand* OR "adverse effect*" OR toxicit* OR medicament* OR "maladie* autoimmun*" OR "maladie* auto-immun*" OR pharmacociné* OR ((Effet* OR événement* OR incident* OR réaction*) AND (indésirable* OR secondaire* OR délétère* OR néfaste* OR noci* OR nuisible*)) OR "thérapie* moléculaire* ciblée*" OR "protéine* de fusion* recombinante*" OR ((affinité* OR monoclona* OR site* OR fix*) AND anticorp*) OR Paratope* OR "Demi-vie" OR "demi vie" OR "modèle* moléculaire*" OR biodisponibilité* OR bio-disponibilité*) in Title Abstract Keyword - (Word variations have been searched)

Caption : The databases were queried February 24th, 2021. Literature published up to and including April 24th, 2021 was considered for evaluation : 12 articles were collected but they were all subsequently excluded based on inclusion criteria defined in the registered Prospero protocol.
End of review April 25th, 2021.
