# Supplementary material for: FcRn as a Transporter for Nasal Delivery of Biologics: A Systematic Review
Source: Int J Mol Sci. 2021 Jun 17;22(12):6475. doi: 10.3390/ijms22126475 (PMC8234196; doi:10.3390/ijms22126475)
Supplement: Supplementary file 1 [file ijms-22-06475-s001.zip › supplementary/Supplementary File S2.pdf]

Supplementary File S2. Detailed Risk of Bias Using OHAT.

Study	Selection bias			Cofounding bias	Performance bias		Attrition bias	Detection bias		Selective reporting bias	Other bias
	Was administered dose or exposure level adequately randomized?	Was allocation to study groups adequately concealed?	Did selection of study participants result in appropriate comparison groups?	Did the study design or analysis account for important confounding and modifying variables?	Were experimental conditions identical across study groups?	Were the research personnel and human subjects blinded to the study group during the study?	Were outcome data complete without attrition or exclusion from analysis?	Can we be confident in the exposure characterizati on?	Can we be confident in the outcome assessment?	Were all measured outcomes reported?	Were there no other potential threats to internal validity ?
Rawool[27], 2008	Definitely High risk of bias	Definitely High risk of bias	Not relevant	Not relevant	Probably high	Definitely high	Probably low	Probably low	Probably low	Probably low	Probably low
Lu[28], 2011	Definitely High risk of bias	Definitely High risk of bias	Not relevant	Not relevant	Definitely Low	Definitely high	Probably low	Definitely low	Probably low	Definitely low	Probably low
Ye[29], 2011	Definitely High risk of bias	Definitely High risk of bias	Not relevant	Not relevant	Definitely Low	Definitely high	Definitely low	Definitely low	Definitely low	Definitely low	Probably low
Bitsaktis[69], 2015	Definitely High risk of bias	Definitely High risk of bias	Not relevant	Not relevant	Definitely Low	Definitely high	Definitely low	Probably low	Probably low	Definitely low	Probably low
Kumar[61], 2018	Definitely High risk of bias	Definitely High risk of bias	Not relevant	Not relevant	Definitely Low	Definitely high	Probably low	Probably low	Probably low	Probably low	Probably high
Bern[26], 2020	Definitely High risk of bias	Definitely High risk of bias	Not relevant	Not relevant	Definitely Low	Definitely high	Definitely low	Probably low	Probably low	Definitely low	Probably low
Rohm[35], 2017	Definitely High risk of bias	Definitely High risk of bias	Not relevant	Not relevant	Definitely Low	Definitely high	Definitely low	Probably low	Probably low	Definitely low	Probably low
Bequignon[9], 2019	Definitely High risk of bias	Definitely High risk of bias	Not relevant	Not relevant	Definitely Low	Definitely high	Definitely low	Probably low	Probably low	Definitely low	Probably low
Ladel[16], 2019	Definitely High risk of bias	Definitely High risk of bias	Not relevant	Not relevant	Probably high	Definitely high	Probably low	Probably low	Probably low	Definitely low	Probably low
Samson[46], 2012	Definitely High risk of bias	Definitely High risk of bias	Not relevant	Not relevant	Probably low	Definitely high	Definitely low	Definitely low	Definitely low	Definitely low	Probably low
Heidl[10], 2015	Definitely High risk of bias	Definitely High risk of bias	Not relevant	Not relevant	Probably low	Definitely high	Probably low	Probably high	Probably low	Probably low	Probably low
Ladel[15], 2018	Definitely High risk of bias	Definitely High risk of bias	Not relevant	Not relevant	Probably high	Definitely high	Probably low	Probably low	Probably low	Definitely low	Probably low
