# Supplementary material for: FcRn as a Transporter for Nasal Delivery of Biologics: A Systematic Review
Source: Int J Mol Sci. 2021 Jun 17;22(12):6475. doi: 10.3390/ijms22126475 (PMC8234196; doi:10.3390/ijms22126475)
Supplement: Supplementary file 1 [file ijms-22-06475-s001.zip › supplementary/Supplementary File S3.pdf]

Supplementary File S3. Type of Samples and Protocols of the Included In Vivo Studies.

First author Date	Design	Species	Age	N	Type of samples and modifications	Protocols
**Rawool**[27]* **2008**	*In vivo*	Mice	6-8 weeks	15-18 mice	**BALB/c and C57BL/6 mice** (Taconic Farms)<br>**IgA−/−** mice (B6.129, Albany Medical College, NY)<br>**FcRn -/-** mice (B6.129×1-Fcrgttm1Dcr/Dcr, the Jackson Laboratory, Bar Harbor, ME)	Day 0 : intranasal immunization with mAb-iFT, iFT or PBS<br>Day 21 : intranasal immunization boost<br>Day 35 : Intranasally challenged with FT LVS or FT SchuS4<br>Survival monitoring during at least 21 days.
**Lu**[28]* **2011**	*In vivo*	Mice	6-8 weeks	20 mice	**C57BL/6 mice** (National Cancer Institute or Charles River)<br>**FcRn -/-** mice (B6.129, the Jackson Laboratory, Bar Harbor, ME)	Day 0 : intranasal immunization with Gag-Fc/wt, Gag-Fc/mut, or Gag-CPG or PBS<br>Day 14 : intranasal immunization boost<br>Day 42 : Intravaginally challenged with HIV-1<br>Day 47 : Ovary were harvested, and virus titers measured<br>Month 4 : IgG antibody titers in serum were measured and splenocytes were isolated
**Ye**[29]* **2011**	*In vivo*	Mice	6-8 weeks	20 mice	**C57BL/6 mice** (National Cancer Institute or Charles River)<br>**FcRn -/-** mice (B6.129, the Jackson Laboratory, Bar Harbor, ME)	Day 0 : intranasal immunization with gD-Fc/WT, gD-Fc/Mut, gD-CPG or PBS<br>Day 14 : intranasal immunization boost<br>Day 50 : Intravaginally challenged with a lethal dose of HSV-2<br>Survival monitoring during at least 15 days.<br>Month 6 : Intravaginally challenged immunized mice with a lethal dose HSV-2
**Bitsatksis**[69]* **2015**	*In vivo*	Mice	8-12 weeks	15-18 mice	**C57BL/6 mice** (Taconic Laboratories, Hudson, NY)<br>**IL-12p35 -/-** mice (B6.129S1-IL12atm1Jm/J, the Jackson Laboratory, Bar Harbor, ME)	Day 0 : intranasal immunization with mAb-iFT, iFT or F(ab')$_2$-iFT<br>Day 21 : intranasal immunization boost<br>Day 35 : Intranasally challenged with FT LVS<br>Survival monitoring during for 21 to 25 days.
**Kumar**[61]* **2018**	*In vivo*	Rats	NA	NA	**Sprague-Dawley rats** (180-200g, Envigo)	Anesthetized and canulated in the abdominal aorta<br>30 minutes after intranasal and intra-arterial delivery<br>- CSF withdrawal, then euthanized for perfusion-fixation of tissue<br>- Blood sampling at 10 minutes intervals<br>- Analysis of IgG quality using SDS-PAGE
**Bern**[26]* **2020**	*In vivo*	Mice	6-8 weeks	30-35 mice	**C57BL/6 and BALB/c mIce**<br>**Tg32 Alb -/-** mice<br>**FcRn -/-** mice and **Alb -/- FcRn -/-** mice<br>**hFcRn +/+ FcRn -/-**<br>(the Jackson laboratory, Bar Harbor, ME)	Pulmonary delivery study and half-life study<br>Day 0 : Preloaded with human albumin or human IgG<br>Day 2 : intranasal delivery of biotinylated albumin (WT, KAHQ or QMP) or IgG1 or scFv-Alb-WT or scFv-QMP<br>-Blood sampling after 4, 8, 24, 96, 168 & 360h (Alb) and 4, 8, 24, 30 & 48h (Alb and IgG1)

Abbreviations: Alb, Albumin; CSF, Cerebro spinal fluid; CPG, CpG ODN1826 (5'-TCCATGACGTTCCTGACGTT-3'; Invivogen, San Diego, CA); FcRn, neonatal Fc Receptor; Gag-Fc, fusion of p24 protein from HIV Gag with IgG heavy chain; hFcRn, human FcRn; HIV, Human immunodeficience virus; IgG, Immunoglobulin G; IL, interleukin; iFt, inactivated Francisella Tularensis; KO, knock-out; LVS, Live vaccine strain; mAb, Monoclonal Antibody; mut, mutant; NA, Not available; PBS, Phosphate Buffer Saline; QMP, triple mutant E505Q/T527M/K573P Albumin engineered; scFv, single-chain variable fragment; SDS-PAGE, sodium dodecyl sulfate polyacrylamide gel electrophoresis; WT, Wild-Type.
