# Supplementary material for: FcRn as a Transporter for Nasal Delivery of Biologics: A Systematic Review
Source: Int J Mol Sci. 2021 Jun 17;22(12):6475. doi: 10.3390/ijms22126475 (PMC8234196; doi:10.3390/ijms22126475)
Supplement: Supplementary file 1 [file ijms-22-06475-s001.zip › supplementary/Supplementary File S4.pdf]

Supplementary File S4. Type of samples and protocols of the included *in vitro* and *ex vivo* studies.

First author Date	Design	Species	Cell density, T°, rH, number of samples	Interface	Type of cells and samples and conditions of culture	Permeation protocols
**Röhm**[35]* **2017**	*In vitro*	RPMI cells	2.10^5 cells per insert 37°C, 5% CO_2	24-well format transwells	Cell line from a carcinoma from squamous epithelium obtained from a human nasal septum cultivated under submerged conditions and under ALI.	Formulated proteins and FITC-dextran were aerosolized above the cells using a nebulization system. After 90 or 240 min incubation time, abluminal medium was harvested to measure biotherapy concentration by either ELISA or fluorescence spectroscopy.
**Bequignon**[9]* **2019**	*In vitro*	HNEC	1.10^6 cells per insert 37°C, pH 6	12-well format transwells	HNECs were isolated from nasal polyps obtained during endoscopic sinus surgery. The cells were cultivated under ALI conditions for 21 days to reach a steady well-differentiated state.	Infliximab was added in the upper chamber. After 4 h, medium from the lower chamber was harvested to measure biotherapy concentration via ELISA.
**Ladel**[16]* **2019**	*In vitro*	OEPC and RPMI cells	1.10^5 cells per insert 37°C, 5% CO_2, 95% rH	NA	OEPC were harvested from mucosal explants from the dorsal part of the *concha nasalis dorsalis* and *medi*. RPMI cells were obtained as described previously[35]. Cells were cultivated under ALI conditions for 21 days.	pIgG and hIgG were added in the upper chamber. After 48 h, 20 µL of the abluminal medium was harvested at 0.5, 2, 4, 8, 12, 24 and 48 h to measure biotherapy concentration via ELISA.
	Ex vivo	Porcine olfactory mucosa	N = 4 35 °C, 90% rH	self-made side-by-side cell system consisting of two 1.5 mL microreaction tubes	Tissue specimens (2 cm^2) were excised from the *concha nasalis media* of 4 to 6 months old slaughterhouse pigs.	pIgG and hIgG were applied on the epithelial layer. After 5 h, mucosa explants were fixed in PFA for immunofluorescence staining and western blotting.
**Samson**[46]* **2012**	*Ex vivo*	Porcine nasal mucosa	N = 7	Mucosal specimens were mounted in vertical static diffusion cells with a surface area of 0.77 cm^2.	Samples from nasal mucosa specimens isolated from porcine snouts obtained from a slaughterhouse.	Surface of the nasal septum and cavity specimen was treated with a single topical exposure of bevacizumab. After 2.5 h, the receptor compartment was harvested every 30 minutes to measure biotherapy concentration via ELISA.
**Heidl**[10]* **2015**	*Ex vivo*	HNEC	N = 7	4µm-thin sections were deparaffinized and rehydrated for antigen retrieval	Samples from the inferior turbinate of patients undergoing routine turbinate surgery were obtained from the department of otorhinolaryngology.	Small pieces of tissue were fixed and paraffin-embedded for immunofluorescence microscopy and western blotting.
**Ladel**[15]* **2018**	*Ex vivo*	Porcine olfactory mucosa	N = 4 35 °C, 90% rH	self-made side-by-side cell system consisting of two 1.5 mL microreaction tubes	Tissue specimens (2 cm^2) were excised from the *concha nasalis dorsalis* and *ventralis* of 4 to 6 months old slaughterhouse pigs.	pIgG and hIgG were applied on the epithelial layer. After 30 min, 2 h, 4 h or 8 h, mucosa explants were fixed in PFA for Immunofluorescence staining and microscopy analysis.

Abbreviations: ALI, Air liquid interface; ELISA, enzyme-linked immunosorbent assay; FcRn, neonatal Fc Receptor; FITC-dextran, Fluorescein isothiocyanate-dextran; HNEC, Human Nasal Epithelial Cell; hIgG, human Immunoglobulin G; NA, Not available; OEPC, primary cells from porcine olfactory epithelium; PFA, paraformaldehyde; pIgG, porcine IgG; RPMI cells, carcinoma from squamous epithelium obtained from a human nasal septum; rH, relative Humidity; T°, Temperature.
